# The Effect of Natural Extracts on Aging and Neurodegenerative Diseases

**DOI:** 10.3390/nu18193125

**Published:** 2026-09-23

**Authors:** Carla Masala, Antonella Rosa

**Affiliations:** Department of Biomedical Sciences, University of Cagliari, Cittadella Universitaria, SP 8 Monserrato, 09042 Cagliari, Italy; anrosa@unica.it

Aging is a complex, multifactorial process characterized by the progressive decline of physiological functions, ultimately leading to increased susceptibility to disease and mortality. This process is closely associated with cognitive decline and impairment of sensory systems, including olfactory and gustatory functions, which significantly compromise the quality of life and functional independence of older adults [1]. Age-related decline is associated with impaired antioxidant defenses and oxidative stress, which may trigger the onset and progression of neurodegenerative diseases (such as Alzheimer’s and Parkinson’s) [2]. There is growing scientific interest in investigating natural extracts with antioxidants and anti-inflammatory properties, driven by the increasing global life expectancy and the rising burden of neurodegenerative diseases. These natural extracts are being extensively explored in both human and animal models for their potential effect in reducing oxidative stress, a key contributor to neuronal damage and disease progression. Consequently, this research aims to identify novel, accessible, and potentially safer natural therapeutic strategies to promote healthy aging and to prevent and manage neurodegenerative conditions. This *Nutrients* Special Issue includes four original studies assessing the role of natural extracts and bioactive molecules in humans, and four investigating their effects in animal models. Oxidative stress is closely related to inflammation, as reactive oxygen species (ROS) are produced in response to inflammatory processes. Among the studies focused on humans, Calzoni et al. (Contribution 1) and Rezaee et al. (Contribution 2) focused on the most common neurodegenerative disease: Alzheimer’s disease. Calzoni et al. evaluated the antioxidant effects of *Rheum rhabarbarum*-derived extracellular vesicles in a cellular model of Alzheimer’s disease, as rhubarb rhizome has emerged as a promising class of natural nanocarriers capable of delivering bioactive phytochemicals with enhanced stability and bioavailability. The *R. rhabarbarum* rhizomes showed antioxidant activity in vitro, reduced intracellular reactive oxygen species, and modulated markers associated with oxidative stress and cellular metabolism in an Alzheimer’s disease cellular model. In detail, the treatment with rhubarb extracellular vesicles decreased intracellular ROS and modulated the expression of oxidative stress-related proteins. Rezaee et al. investigated whether polyphenolic extracts from six sorghum varieties can counteract Alzheimer’s disease-related cellular abnormalities, such as tau hyperphosphorylation and mitochondrial dysfunction. The sorghum extracts reduced Aβ-induced tau abnormalities and improved mitochondrial readouts in a dose-dependent manner; in particular, they reduced beta-amyloid-induced phospho-tau levels, restored mitochondrial membrane potential, and increased ATP levels. 

The study by Hanyuda et al. (Contribution 3) focused on glaucoma, an age-related neurodegenerative disease characterized by progressive retinal ganglion cell degeneration and visual decline. The authors reported that a multi-nutrient supplement containing red ginger (*Zingiber officinale*) extract was associated with acute, short-term increases in optic nerve head blood flow in patients with open-angle glaucoma. These data suggest that red ginger may induce vasodilation and exert neuroprotective effects, thereby supporting ocular perfusion in glaucoma.

Focused on preventing the onset of mild cognitive impairment and Alzheimer’s disease, the study by Rosa et al. (Contribution 4) showed that olfactory function could be a potential early indicator of attention, memory, language, and visuospatial/executive dysfunctions. This study evaluated the potential effect of *Rosmarinus officinalis* (rosemary) and *Carum carvi* (caraway) in relation to aging. Rosemary and caraway EOs were perceived without significant decreases in odor pleasantness, intensity, and familiarity ratings across age groups, suggesting their potential bioactive effects in promoting healthy aging.

Among the studies using animal models, Tranchida et al. (Contribution 5) investigated whether supplementation with *Vitis vinifera* leaf extract, a polyphenol-rich natural formulation containing resveratrol, catechins, and anthocyanins, may reduce oxidative stress and stabilize tight junction proteins in the blood–brain barrier and in the intestinal barriers after traumatic brain injury (TBI) in mice. The authors support the idea that polyphenol-rich nutritional interventions may offer a multi-target strategy for reducing inflammation both in brain and intestinal tissues after TBI.

The other studies evaluated the effects of natural extracts and bioactive molecules in the progression and development of neurodegenerative diseases in animal models.

Cui et al. (Contribution 6) provided preliminary in vitro evidence in mouse-derived microglia supporting the anti-neuroinflammatory effect of dendrobine, a bioactive molecule product of *Dendrobium*, known for its neuroprotective activity. The findings indicate that dendrobine suppresses inflammatory activation, reduces oxidative stress, and could be used in the prevention of neuroinflammation-related disorders.

The study by Hack et al. (Contribution 7) evaluated the effects of triterpene and caffeoylquinic acids found in the water extract of *Centella asiatica* in aged mice. *C. asiatica* is an edible plant used in traditional medicine for its effects on learning, cognitive function, and memory. The authors suggested that the triterpene and caffeoylquinic acids may improve age-related cognitive changes, especially learning and cognitive function.

In addition, the study by Ramirez-Sanchez et al. (Contribution 8) evaluated the effects of (+)-Epicatechin, a flavanol found in cacao, on short- and long-term memory in aged rats by assessing protein carbonyls and malondialdehyde levels. These data indicated that (+)-Epicatechin may enhance mitochondrial function, reduce oxidative stress and inflammation in the hippocampus, and improve short- and long-term memory in aged rats. 

In conclusion, this Special Issue, combining preclinical and clinical studies, provides a solid rationale for investigating the effects of natural extracts on aging and neurodegenerative diseases. Collectively, these contributions underscore the growing value of natural compounds as promising tools for modulating the biological pathways in age-related decline and neurodegeneration.
